# Human Cytomegalovirus UL7, miR-US5-1, and miR-UL112-3p Inactivation of FOXO3a Protects CD34^+^ Hematopoietic Progenitor Cells from Apoptosis

**DOI:** 10.1128/mSphere.00986-20

**Published:** 2021-01-06

**Authors:** Meaghan H. Hancock, Lindsey B. Crawford, Wilma Perez, Hillary M. Struthers, Jennifer Mitchell, Patrizia Caposio

**Affiliations:** a Vaccine and Gene Therapy Institute, Oregon Health & Science University, Portland, Oregon, USA; University of North Carolina, Chapel Hill

**Keywords:** FOXO3a, apoptosis, hematopoietic progenitor cells, human cytomegalovirus

## Abstract

Human cytomegalovirus (HCMV) infection of myeloid lineage cells, such as CD34^+^ hematopoietic progenitor cells (HPCs) or monocytes, results in the upregulation of antiapoptotic cellular proteins that protect the newly infected cells from programmed cell death. The mechanisms used by HCMV to regulate proapoptotic cellular proteins upon infection of CD34^+^ HPCs have not been fully explored. Here, we show that HCMV utilizes pUL7, a secreted protein that signals through the FLT3 receptor, and miR-US5-1 and miR-UL112-3p to reduce the abundance and activity of the proapoptotic transcription factor FOXO3a at early times after infection of CD34^+^ HPCs. Regulation of FOXO3a by pUL7, miR-US5-1, and miR-UL112 results in reduced expression of the proapoptotic *BCL2L11* transcript and protection of CD34^+^ HPCs from virus-induced apoptosis. These data highlight the importance of both viral proteins and microRNAs (miRNAs) in protecting CD34^+^ HPCs from apoptosis at early times postinfection, allowing for the establishment of latency and maintenance of viral genome-containing cells.

**IMPORTANCE** Human cytomegalovirus (HCMV) causes serious disease in immunocompromised individuals and is a significant problem during transplantation. The virus can establish a latent infection in CD34^+^ hematopoietic progenitor cells (HPCs) and periodically reactivate to cause disease in the absence of an intact immune system. What viral gene products are required for successful establishment of latency is still not fully understood. Here, we show that both a viral protein and viral miRNAs are required to prevent apoptosis after infection of CD34^+^ HPCs. HCMV pUL7 and miRNAs miR-US5-1 and miR-UL112-3p act to limit the expression and activation of the transcription factor FOXO3a, which in turn reduces expression of proapoptotic gene *BCL2L11* and prevents virus-induced apoptosis in CD34^+^ HPCs.

## Abstract (precis)

Human cytomegalovirus (HCMV) causes serious disease in immunocompromised individuals and is a significant problem during transplantation. The virus can establish a latent infection in CD34^+^ hematopoietic progenitor cells (HPCs) and periodically reactivate to cause disease in the absence of an intact immune system.

## INTRODUCTION

Human cytomegalovirus (HCMV) is prevalent in the majority of the world’s population and results in a lifelong persistence with periods of latency and reactivation. Primary infection in healthy individuals is typically subclinical and is controlled by the host immune response. However, HCMV infection of immunocompromised hematopoietic stem cell (HSCT) or solid organ (SOT) transplant recipients remains a major cause of life-threatening disease even with the development of antiviral therapy (1–3). The main source of latent HCMV is hematopoietic progenitor cells (HPCs) and myeloid lineage cells (4–6). HCMV expresses a limited set of genes during latency including US28, transcriptional variants from the UL133-138 region, LUNA, a subset of viral microRNAs (miRNAs), and the β2.7 long noncoding RNA (lncRNA). Viral reactivation from latency is tightly linked to myeloid differentiation that results in the expression of all the genes necessary to produce infectious virus in macrophages (7, 8). The HCMV latency genes function through modulation of cellular processes necessary for maintenance of the viral genome in the cell. The triggers for viral reactivation and the viral and cellular genes that mediate this process are largely unknown.

HCMV infection of cells results in the activation of proapoptotic cell death pathways that are detrimental to viral replication. HCMV encodes a number of antiapoptotic proteins that function during lytic infection to block this cellular response. These HCMV antiapoptotic proteins include pUL36 (vICA) that interacts with procaspase-8 to inhibit proteolytic processing (9), pUL37 (vMIA) that sequesters proapoptotic BAX at the outer mitochondrial membrane and prevents cytochrome *c* release (10), and the pUL38 gene product that blocks proteolysis of two key apoptotic enzymes, caspase-3 and poly(ADP-ribose) polymerase (PARP) (11). In addition to these proteins, the HCMV immediate early (IE) protein IE2 was observed to upregulate the protease-deficient procaspase-8 homologue, c-FLIP, that decreases the activities of caspase-3 and caspase-8 (12). Also, HCMV lncRNA β2.7 was shown to interact with the mitochondrial enzyme complex I to stabilize mitochondrial membrane potential and prevent apoptotic death of HCMV-infected neuronal cells (13). Several HCMV miRNAs have also been described that protect cells from apoptosis during lytic infection. HCMV miR-UL36-5p inhibits apoptosis via downregulation of adenine nucleotide translocator 3, an adenine nucleotide transporter responsible for translocating ADP and ATP across the mitochondrial membrane (14). Additionally, miR-UL148D protects cells from apoptosis induced by overexpression of IEX-1 (15). Finally, Kim et al. reported *FAS* as a target of miR-UL36-3p, miR-US5-1, and miR-US5-2-3p; *Fas associated protein with death domain (FADD)* as a target of miR-US5-1; *Caspase-3* as a target of miR-US25-2-3p, miR-112-5p, and miR-UL22A-5p; *Caspase-2* as a target of miR-US4-5p; and *Caspase-7* as a target of miR-UL22A-3p and miR-US4-3p (16).

In nonpermissive cells (CD34^+^ HPCs), or in cells with a protracted life cycle (monocytes), early survival of HCMV-infected cells is achieved by virus-induced regulation of antiapoptotic cellular factors such as myeloid cell leukemia (MCL)-1 and B cell lymphoma-2 (Bcl-2) (reviewed in reference 17). In monocytes, the binding of HCMV gB to epidermal growth factor receptor (EGFR), as well as the binding of the viral pentameric complex to integrins, drives signaling through phosphatidylinositol 3-kinase (PI3K) and mTOR kinase that leads to the upregulation of MCL-1 (18). The virus, after 48 h of infection, shifts from MCL-1 to Bcl-2 as the primary antiapoptotic tool, and the upregulation of Bcl-2 is mediated by integrin signaling events following initial viral binding (19). In CD34^+^ HPCs, Reeves et al. showed that MCL-1 is upregulated via gB stimulation of mitogen-activated protein kinase (MAPK) signaling in the absence of *de novo* viral gene expression (20). Furthermore, the protective phenotype driven by the virus-induced extracellular signal-regulated kinase (ERK)-MAPK signaling correlates with the downregulation of the proapoptotic proteins PUMA and BIM. At the same time, ERK-MAPK signaling phosphorylates the transcription factor ELK-1 that is required for MCL-1 expression and cell survival (21).

Expression of cellular pro- and antiapoptotic genes is carefully regulated to allow for the timely elimination of transformed and virus-infected cells. The mammalian Forkhead Box O (FOXO) family of transcription factors, including FOXO1, FOXO3a, FOXO4, and FOXO6, is implicated in a wide variety of physiologic processes such as cell cycle arrest, cellular differentiation, DNA repair, and cell death (reviewed in references 22 and 23). The FOXO family of transcription factors promotes apoptosis by mitochondrion-dependent and -independent mechanisms, including mediating expression of the Bcl-2-like protein 11 (BIM), a proapoptotic Bcl-2 family protein (24). FOXO proteins are normally present in an active state in the cell’s nucleus. Upon growth factor stimulation, they are phosphorylated by different serine/threonine cellular kinases triggering inactivation and export of FOXOs from the nucleus to the cytoplasm (25; reviewed in reference 26).

HCMV UL7 is part of the RL11 gene family and is dispensable for lytic viral replication (27). Our group and others have shown that UL7 is a transmembrane glycoprotein that is secreted from infected cells (27–29). Among clinical and lab-adapted HCMV strains, UL7 sequence is very well conserved (97 to 100% intragenotype conservation and 83 to 93% intergenotype conservation), suggesting a crucial role in viral replication in the host (30). Our group recently demonstrated that HCMV pUL7 is a ligand for the cytokine receptor Fms-like tyrosine kinase 3 (Flt-3R) (31). Signaling through the Flt-3R is critical for normal development of hematopoietic stem and progenitor cells (32). We observed that pUL7 binding to Flt-3R induces activation of the downstream PI3K/AKT and MAPK/ERK signaling pathways. Importantly, we have shown that UL7 protein induces both CD34^+^ HPCs and monocyte differentiation *in vitro* and *in vivo* and thus functions as a hematopoietic differentiation factor (31). Although UL7 is nonessential for lytic replication, HCMV UL7 mutants fail to reactivate from latency in CD34^+^ HPCs (31).

In the current study, we observed that pUL7 signaling via Flt-3R promotes a rapid phosphorylation of FOXO3a specifically through the MAPK pathway. The phosphorylation of FOXO3a results in nuclear-to-cytoplasmic translocation and inactivation of the transcription factor as demonstrated by the downregulation of the FOXO3a target gene *BCL2L11*. Additionally, we show that HCMV miR-US5-1 and miR-UL112-3p directly reduce FOXO3a transcript and protein levels, resulting in reduced *BCL2L11* mRNA expression, indicating that the virus utilizes multiple mechanisms to modulate FOXO3a activity. Finally, we observed that UL7, miR-US5-1, and miR-UL112-3p are expressed in the early stages of HCMV infection in CD34^+^ HPCs and act to reduce FOXO3a activity to promote survival of infected hematopoietic progenitor cells.

## RESULTS

### pUL7 signaling promotes phosphorylation of FOXO3a via the MAPK pathway.

**FIG 1:**
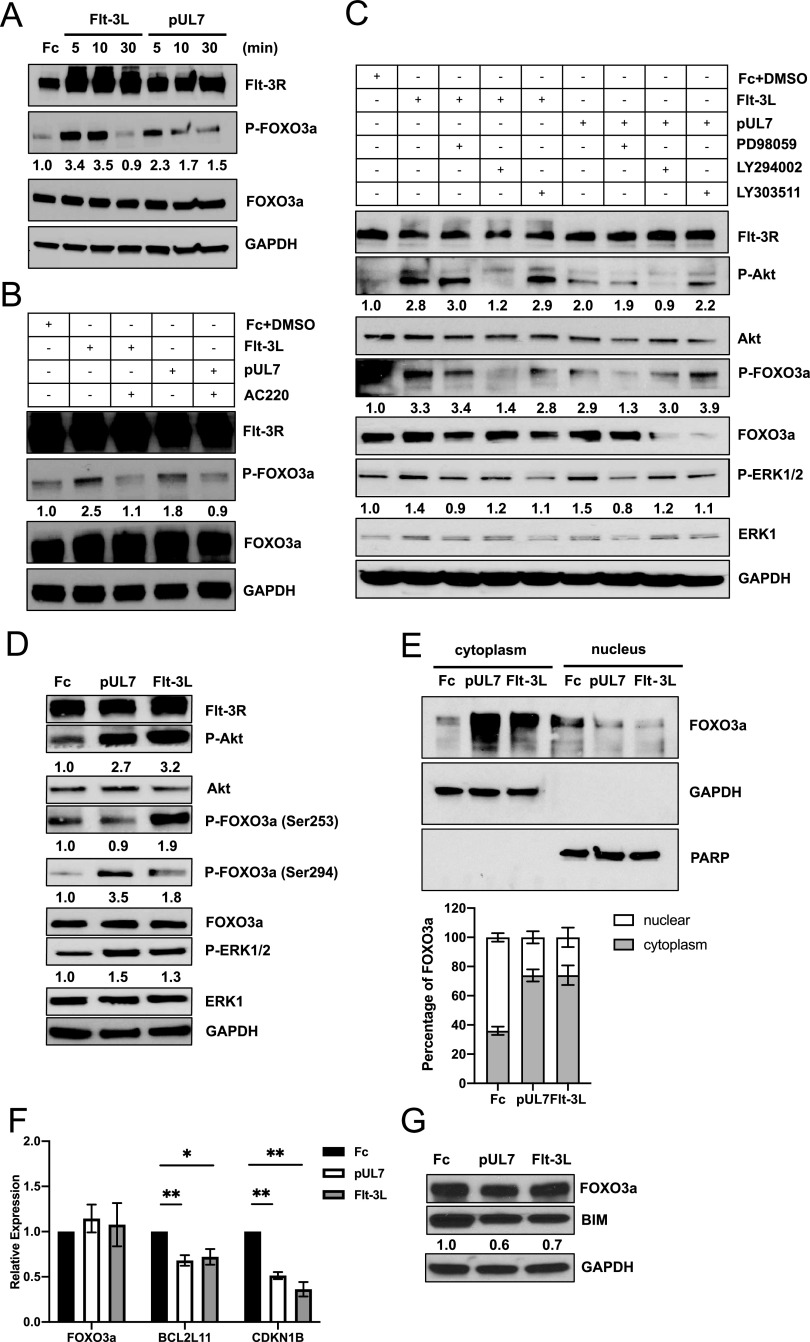
pUL7 promotes FOXO3a phosphorylation via MAPK pathway. (A) Protein lysates were generated and immunoblotted for phosphorylation of FOXO3a from serum-starved THF-Flt-3R stimulated with 50 ng/ml of Fc, pUL7, or Flt-3L for the indicated time. (B) THF-Flt-3R cells were pretreated with AC220 (100 nM) for 1 h and then stimulated for 10 min with Fc + DMSO, pUL7, or Flt-3L. Protein lysates were generated and immunoblotted for phosphorylation of FOXO3a (Ser253/Se294). (C) THF-Flt-3R cells were pretreated with PD98059 (50 μM), LY294002 (20 μM), or LY303511 (25 μM) for 1 h and then stimulated for 10 min. Protein lysates were generated and immunoblotted for phosphorylation of Akt (Thr308), ERK1/2 (Thr202/Tyr204), or FOXO3a. (D) Protein lysates were generated from THF-Flt-3R stimulated for 10 min and immunoblotted for phosphorylation of Akt (Thr308), ERK1/2 (Thr202/Tyr204), FOXO3a (Ser253), and FOXO3a (Ser294). Equal loading was confirmed by Flt-3R, Akt, ERK1, FOXO3a, and GAPDH antibody staining. Numbers under the immunoblots indicate relative phosphorylation of FOXO3a, Akt, or ERK1/2 normalized to the amount of total FOXO3a, Akt, or ERK1 and compared to the value of the Fc control. (E) Nuclear versus cytoplasmic lysates were generated from THF-Flt-3R stimulated for 10 min and immunoblotted for total FOXO3a. GAPDH and PARP were used as cytosolic and nuclear markers, respectively. Relative intensity was quantified by densitometry and normalized to the loading controls. The percentage of cytoplasmic and nuclear FOXO3a was determined as follows: cytoplasmic FOXO3a density/(cytoplasmic + nuclear FOXO3a density) × 100 and nuclear FOXO3a density/(cytoplasmic + nuclear FOXO3a density) × 100, respectively. Results are representative of three independent experiments. (F) Serum-starved THF-Flt-3R cells were stimulated for 24 h with Fc, pUL7, or Flt-3L. RNA was isolated using TRIzol, and qRT-PCR for *FOXO3a* and the downstream targets *BCL2L11* and *CDKN1B* was performed. Values are means ± standard errors of the means (SEM) (error bars) from three independent experiments. Statistical significance was determined by unpaired Student’s *t* test (*, *P* < 0.05; **; *P* < 0.005; ***, *P* < 0.0005). (G) Analysis of impact of pUL7 and Flt-3L on BIM protein levels. THF-Flt-3R cells were stimulated as described for panel F, after 24-h protein lysates were immunoblotted for FOXO3a and BIM. Relative BIM intensity was quantified by densitometry and normalized to the GAPDH loading control.

We recently demonstrated that pUL7 binds and signals through the Flt-3R (31). Flt-3 ligand (Flt-3L) induction of AKT/PKB activation was reported to lead to phosphorylation and inactivation of FOXO3a (33), but since MAPK/ERK signaling can also lead to FOXO3a phosphorylation (34), we first determined if pUL7 was able to promote phosphorylation of FOXO3a and which signaling pathway was involved in this process. Since few primary cell types express the Flt-3R, we established Flt-3L-responsive cells by retroviral gene transfer of the human *Flt-3R* gene into telomerized human fibroblasts (THF). When THF-Flt-3R cells were stimulated by pUL7 or the control Flt-3L, FOXO3a was rapidly phosphorylated in a time-dependent manner (Fig. 1A; see also Fig. S1A in the supplemental material) and the response was dependent on the Flt-3R as demonstrated by the treatment with the Flt-3R inhibitor AC220 (Fig. 1B and Fig. S1B). To determine which Flt-3R downstream signaling pathway was involved in FOXO3a phosphorylation we stimulated the cells in the presence of a PI3K inhibitor (LY294002) or a MEK inhibitor (PD98059), and we used as a control an S6K inhibitor (LY303511) to rule out off-target effects. As previously reported, phosphorylation of FOXO3a by Flt-3L was PI3K dependent as addition of LY294002 significantly decreased the level of FOXO3a phosphorylation, while pUL7 resulted in phosphorylation of FOXO3a through the MAPK pathway. Indeed, neither LY294002 nor LY303511 had any effect on pUL7-mediated FOXO3a phosphorylation; only the MAP kinase inhibitor PD98059 was able to prevent pUL7-induced FOXO3a phosphorylation, as supported by statistical analysis of densitometric data (Fig. 1C and Fig. S1C).

10.1128/mSphere.00986-20.1 FIG S1 Quantification of protein expression of Fig. 1A to D. Relative protein expression of three independent Western blotting assays was quantified using Image J. *, *P* < 0.05, and **, *P* < 0.005, compared to THF-Flt-3R cells stimulated with Fc or Fc + DMSO. Download FIG S1, EPS file, 1.5 MB. Copyright © 2021 Hancock et al. 2021 Hancock et al. This content is distributed under the terms of the Creative Commons Attribution 4.0 International license.

To further confirm this observation, we used specific phospho-FOXO3a antibodies that recognize serine residues phosphorylated by Akt (Ser253) or ERK1/2 (Ser294) (26). As shown in Fig. 1D, stimulation with Flt-3L induced significant phosphorylation of FOXO3a on Ser253, while pUL7 stimulation resulted in FOXO3a phosphorylation on Ser294 (Fig. S1D). Overall, FOXO3a levels were lower in the cytoplasm of the control (35.99% ± 2.89%) than in the Flt-3L (74.05% ± 6.66%)- and pUL7 (73.91% ± 4.12%)-stimulated cells (Fig. 1E), further supporting the observation that FOXO3a is inactivated by pUL7.

As a transcription factor, FOXO3a has been shown to regulate cell death through several downstream targets, including *BCL2L11* (BIM) and *CDKN1B* (p27) (35). Indeed, THF-Flt-3R cells treated with pUL7 or Flt-3L for 24 h have significantly lower levels of *BCL2L11* (pUL7, *P* = 0.0054; Flt-3L, *P* = 0.0325) and *CDKN1B* (pUL7, *P* = 0.0021; Flt-3L, *P* = 0.0013) compared to the control (Fig. 1F). We further analyzed the impact of pUL7 and Flt-3L on BIM protein levels, and as shown in Fig. 1G, in cells stimulated with pUL7 or Flt-3L we observed a reduction in protein expression. Finally, stimulation with pUL7 or Flt-3L did not affect FOXO3a mRNA or protein expression, confirming that pUL7 regulates FOXO3a via posttranslational modifications without affecting mRNA or total protein levels (Fig. 1F and G).

### pUL7 promotes cytoplasmic translocation of FOXO3a and downregulation of *BCL2L11* in myeloid cells.

**FIG 2:**
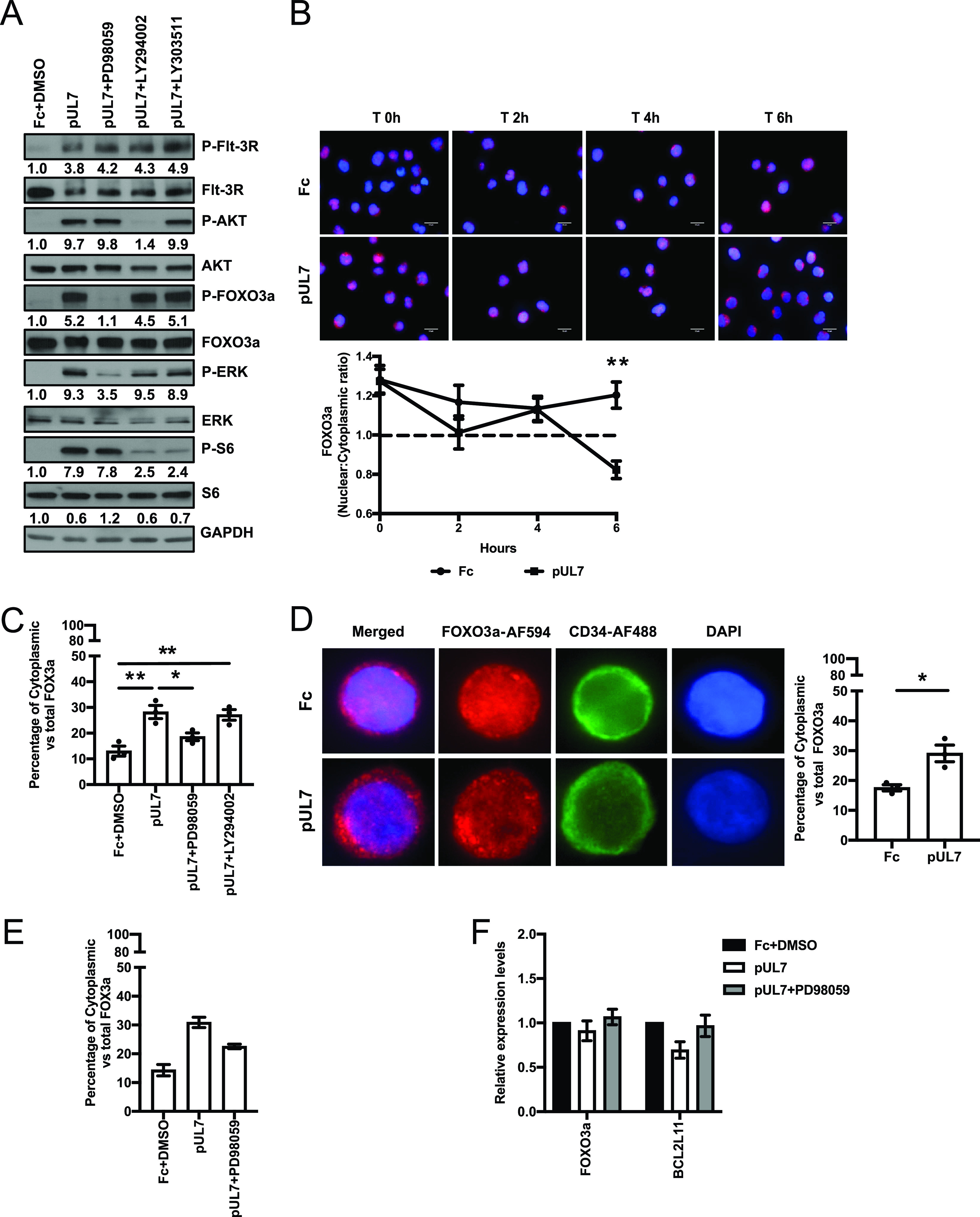
pUL7 induces FOXO3a translocation from the nucleus to the cytoplasm in RS4;11 and CD34^+^ HPC cells. (A) RS4;11 cells were serum starved; pretreated with PD98059 (50 μM), LY294002 (20 μM), or LY303511 (25 μM) for 1 h; and then stimulated for 10 min with Fc + DMSO or pUL7. Protein lysates were generated and immunoblotted for phosphorylation of Flt-3R (Tyr591), Akt (Thr308), ERK1/2 (Thr202/Tyr204), FOXO3a (Ser 294), and S6 (Ser235/236). Equal loading was confirmed by Flt-3R, Akt, ERK1, FOXO3a, S6, and GAPDH antibody staining. Numbers under the immunoblots indicate relative phosphorylation of Flt-3R, Akt, FOXO3a, ERK1/2, or S6 normalized to the amount of total Flt-3R, Akt, FOXO3a, ERK1, and S6 and compared to the value of the Fc control. (B) Subcellular localization of FOXO3a in RS4;11 cells stimulated with Fc and pUL7 (50 ng/ml) at the indicated time points (representative images). FOXO3a (red, secondary antibody conjugated to AF594) and nucleus (DAPI, blue). Images were quantified using ImageJ, and the ratio of nuclear to cytoplasmic FOXO3a over time is shown. The dashed line indicates transition from higher signal in the nucleus (above) to cytoplasm (below). An average of 30 cells was analyzed per condition. Values are means ± standard errors of the means (SEM) (error bars) from three independent experiments. Statistical significance was determined by unpaired Student’s *t* test (*, *P* < 0.05; **, *P* < 0.005; ***, *P* < 0.0005). (C) RS4;11 cells were serum starved, pretreated with PD98059 (50 μM) or LY294002 (20 μM) for 1 h, and then stimulated for 6 h with pUL7 (50 ng/ml). Fc + DMSO was used as a control. Subcellular localization of FOXO3a was quantified as described in panel B. An average of 30 cells was analyzed per condition. Values are means ± standard errors of the means (SEM) (error bars) from three independent experiments. Statistical significance was determined by one way-ANOVA with Tukey’s *post hoc* test (*, *P* < 0.05; **, *P* < 0.005; ***, *P* < 0.0005). (D) Bone marrow CD34^+^ HPCs were stimulated for 6 h with Fc or pUL7 (50 ng/ml) and then stained with anti-FOXO3a antibody and Alexa Fluor AF594-conjugated secondary antibody to detect FOXO3a (red) and with anti-CD34 antibody and Alexa Fluor AF488-conjugated secondary antibody to detect CD34 (green). DAPI was used to counterstain the nucleus (blue). Quantification was done comparing percentages of cytoplasmic to the total FOXO3a. An average of 30 cells was analyzed per condition. Values are means ± standard errors of the means (SEM) (error bars) from three independent donors. Statistical significance was determined by unpaired Student’s *t* test (*, *P* < 0.05; **, *P* < 0.005; ***, *P* < 0.0005). (E) CD34^+^ HPCs were pretreated with PD98059 (10 μM) for 1 h, stimulated for 6 h with Fc + DMSO or pUL7 (50 ng/ml), and then stained with anti-FOXO3a antibody and Alexa Fluor AF594-conjugated secondary antibody to detect FOXO3a (red) and with anti-CD34 antibody and Alexa Fluor AF488-conjugated secondary antibody to detect CD34 (green). DAPI was used to counterstain the nucleus (blue). Quantification was done as described in panel D. Values are means ± standard errors of the means (SEM) (error bars) from two independent donors. (F) CD34^+^ HPCs were pretreated with PD98059 (10 μM) for 1 h and then stimulated for 6 h with Fc + DMSO or pUL7 (50 ng/ml). RNA was isolated using TRIzol, and qRT-PCR for *FOXO3a* and the downstream target gene *BCL2L11* was performed. Values are means ± standard errors of the mean (SEM) (error bars) from two independent donors.

To validate our findings in myeloid cells, we stimulated Flt-3R-expressing bone marrow lymphoblast RS4;11 cells with pUL7 in the presence or absence of different chemical inhibitors. As previously observed, pUL7-mediated phosphorylation of FOXO3a was inhibited by the MEK inhibitor (PD98059), but not by the PI3K inhibitor (LY294002) or the control S6K inhibitor (LY303511) (Fig. 2A). When we examined how inactivation of FOXO3a affects the distribution of protein between the cytoplasm and nucleus, we found that in Fc-treated cells FOXO3a was mostly nuclear at each time point posttreatment. However, upon pUL7 stimulation, FOXO3a translocated into the cytoplasm, with significantly less FOXO3a in the nucleus after 6 h (*P* = 0.0033, Fig. 2B). To demonstrate that FOXO3 phosphorylation is directly required for cytoplasmic translocation, RS4;11 cells were pretreated with PD98059 or LY294002 and then stimulated with pUL7. As shown in Fig. 2C, only inhibition of MEK significantly decreased the percentage of cytoplasmic FOXO3 compared to the pUL7-untreated control (*P* = 0.0422). Next, we analyzed FOXO3a translocation in primary CD34^+^ HPCs, and as shown in Fig. 2C, we observed that FOXO3a protein is more abundant in the cytoplasm of pUL7-stimulated cells than in the control (*P* = 0.0179). To confirm the relationship between ERK-mediated FOXO3a phosphorylation and nuclear translocation in CD34^+^ HPCs, two independent donors were pretreated with the PD98059 inhibitor before stimulation with pUL7. As shown in Fig. 2E, inhibition of ERK reduced FOXO3 cytoplasmic translocation promoted by pUL7. Finally, we observed that upon pUL7 stimulation *BCL2L11* transcript levels were decreased compared to the Fc control and treatment with the MEK inhibitor restored *BCL2L11* mRNA levels (Fig. 2F).

Taken together, these data demonstrate that pUL7 plays a pivotal role in the ERK-mediated phosphorylation and translocation of FOXO3a from the nucleus to the cytoplasm in progenitors and myeloid cells, leading to inactivation of the transcription factor and reduction in expression of the proapoptotic gene *BCL2L11*.

### HCMV miR-US5-1 and miR-UL112 cooperatively target FOXO3a.

**FIG 3:**
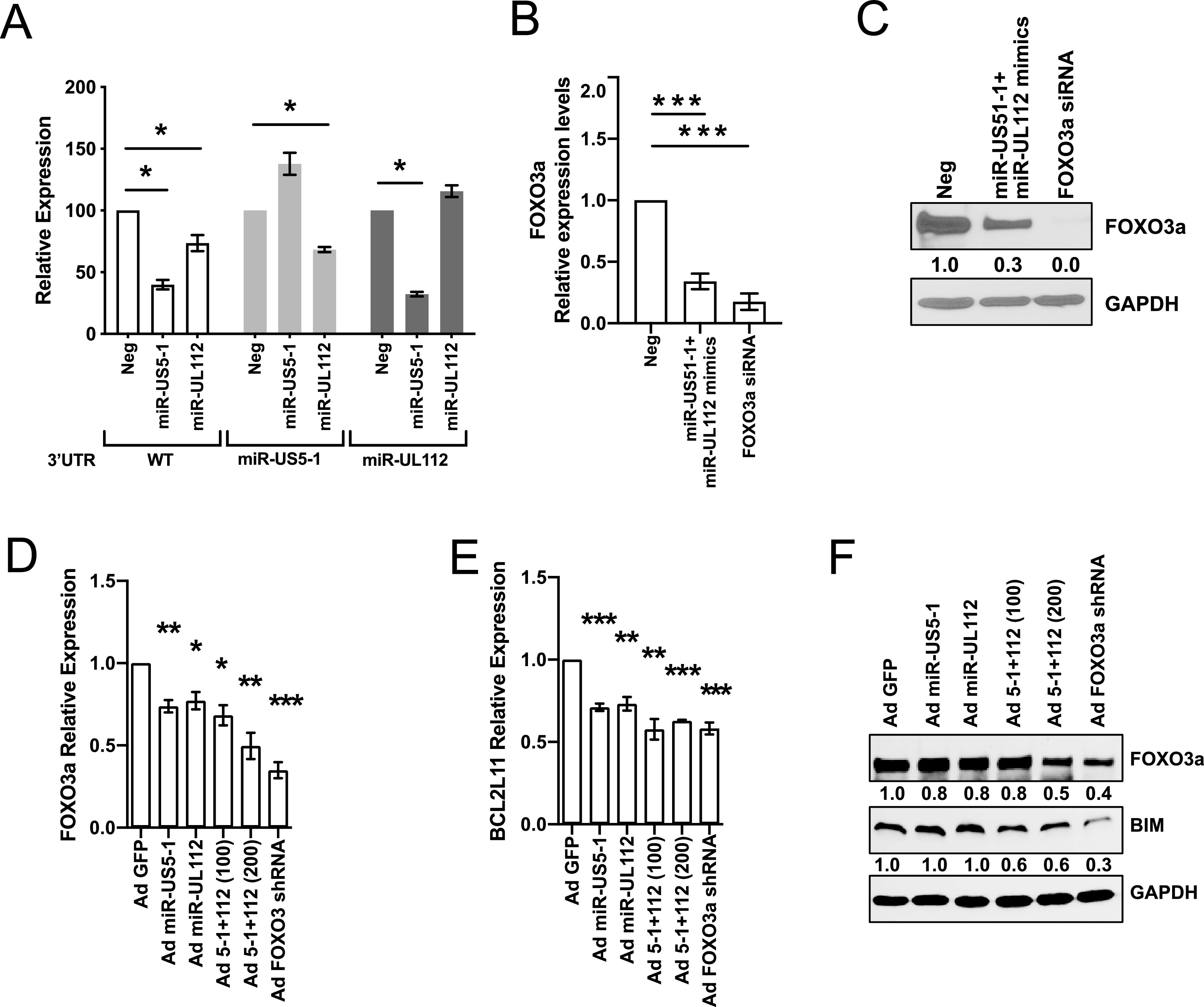
HCMV miR-US5-1 and miR-UL112-3p target FOXO3a protein for downregulation. (A) The wild-type (WT) FOXO3a 3′ UTR or 3′ UTRs containing mutations in the miR-US5-1 or miR-UL112 binding sites were cloned into the pSiCheck2 dual luciferase vector. HEK293T cells were transfected with the pSiCheck2 vector along with negative-control miRNA or miR-US5-1 or miR-UL112-3p mimic. Twenty-four hours later, cells were lysed and luciferase was measured. Values are means ± standard errors of the means (SEM) (error bars) for three independent experiments. Statistical significance was determined by unpaired Student’s *t* test (*, *P* < 0.05; **, *P* < 0.005; ***, *P* < 0.0005). (B) Normal human dermal fibroblasts (NHDF) were transfected with negative-control miRNA and miR-US5-1 and miR-UL112-3p mimics or a FOXO3a small interfering RNA (siRNA) for 48 h. RNA was isolated using TRIzol, and qRT-PCR for *FOXO3a* was performed. Values are means ± standard errors of the means (SEM) (error bars) for three independent experiments. Statistical significance was determined by unpaired Student’s *t* test (*, *P* < 0.05; **, *P* < 0.005; ***, *P* < 0.0005). (C) NHDF were transfected as in panel B, and protein was subjected to immunoblotting. Images were quantified using ImageJ and the ratio of FOXO3a to GAPDH protein, normalized to negative (Neg) control. (D and E) NHDF were serum starved and then transduced with the different adenoviral vectors for 48 h at an MOI of 200 or 100 when in combination. RNA was isolated using TRIzol, and qRT-PCR for *FOXO3a* (D) or *BCL2L11* (E) was performed. Values are means ± standard errors of the means (SEM) (error bars) for three independent experiments. Statistical significance was determined by unpaired Student’s *t* test (*, *P* < 0.05; **, *P* < 0.005; ***, *P* < 0.0005). (F) NHDF were treated as described in panel D, and protein was subjected to immunoblotting. Images were quantified using ImageJ and the ratio of FOXO3a and BIM to GAPDH protein, normalized to Ad GFP control.

There are numerous examples of redundancy between herpesvirus proteins and miRNAs (36, 37). For example, FOXO3a is targeted for inactivation by both gammaherpesvirus proteins and miRNAs (38). Thus, we investigated whether FOXO3a could also be a target of HCMV miRNAs. Biochemical and bioinformatic analysis (16) suggested that FOXO3a was a target of HCMV miR-US5-1 and miR-UL112-3p. We cloned the 3′ untranslated region (UTR) of FOXO3a or 3′ UTRs containing deletions of the potential miR-US5-1 or miR-UL112-3p target sites into a dual luciferase vector. HEK293T cells were transfected with the luciferase vectors along with negative-control miRNA or miR-US5-1 or miR-UL112-3p miRNA mimics. Both miR-US5-1 and miR-UL112-3p target the 3′ UTR of FOXO3a through the identified sites (Fig. 3A). The HCMV miRNAs are capable of downregulating FOXO3a transcript (*P* < 0.0001, Fig. 3B) and endogenous protein (*P* = 0.046, Fig. 3C) after transfection of miRNA mimics into human fibroblasts. While FOXO3a protein levels were somewhat reduced in cells transfected with miR-US5-1 or miR-UL112-3p miRNA mimics alone, the reduction did not reach statistical significance (Fig. S2A). Since transfection efficiency in CD34^+^ HPCs is usually low, we generated green fluorescent protein (GFP)-expressing adenoviral vectors additionally expressing miR-US5-1, miR-UL112-3p, and/or a FOXO3a short hairpin RNA (shRNA) to validate our results in progenitor cells. The other advantage of using the adenovirus (Ad) system is the presence of a GFP expression cassette that allows for sorting of a pure population of transduced cells. We first validated the function of Ad miR-US5-1, Ad miR-UL112-3p, and Ad FOXO3a shRNA in human fibroblasts. Expression of either the two HCMV miRNAs or the control shRNA significantly downregulates *FOXO3a* (Fig. 3D) and *BCL2L11* mRNA (Fig. 3E) as well as FOXO3a and BIM protein levels (Fig. 3F and Fig. S2B). These results clearly demonstrate that FOXO3 is a target of HCMV miR-US5-1 and UL112-3p.

10.1128/mSphere.00986-20.2 FIG S2 Quantification of protein expression of Fig. 3C and F. Relative protein expression of three independent Western blotting assays was quantified using Image J. *, *P* < 0.05, and **, *P* < 0.005, compared to fibroblasts transfected with the mimic negative control (Fig. 3C) or transduced with Ad GFP (Fig. 3F). Download FIG S2, EPS file, 1.3 MB. Copyright © 2021 Hancock et al. 2021 Hancock et al. This content is distributed under the terms of the Creative Commons Attribution 4.0 International license.

### Expression of HCMV UL7, miR-US5-1, and miR-UL112-3p protects CD34^+^ HPCs from apoptosis.

**FIG 4:**
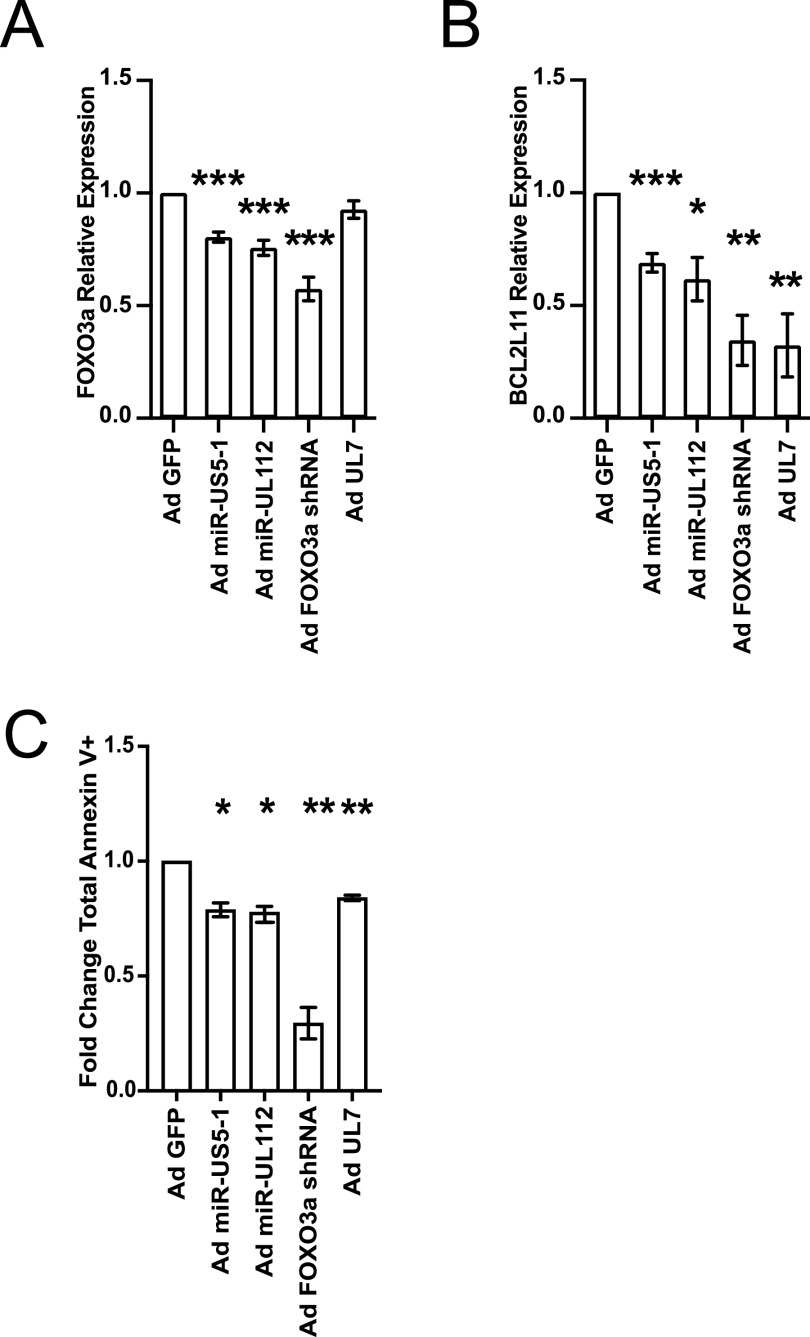
HCMV UL7, miR-US5-1, and miR-UL112-3p protect CD34^+^ HPCs from apoptosis. CD34^+^ HPCs were transduced at an MOI of 500 with Ad GFP, Ad miR-US5-1, Ad miR-UL112-3p, Ad FOXO3a shRNA, or Ad UL7 for 48 h and then FACS isolated for viable CD34^+^ GFP^+^ HPCs. (A and B) RNA was isolated using TRIzol, and qRT-PCR for *FOXO3a* (A) and *BCL2L11* (B) was performed. Values are means ± standard errors of the means (SEM) (error bars) compared to Ad GFP for three independent CD34^+^ donors. Statistical significance was determined by unpaired Student’s *t* test (*, *P* < 0.05; **, *P* < 0.005; ***, *P* < 0.0005). (C) CD34^+^ HPCs were transduced as described above and analyzed by flow cytometry for apoptosis induction after 72 h. The total population was gated on total single, CD34^+^ GFP^+^ HPCs and analyzed for annexin V^+^ (early apoptotic cells) plus annexin V^+^ and viability dye^+^ (late apoptotic and dead cells). The fold change in total annexin V^+^ cells compared to the Ad GFP group for three independent donors is shown. Statistical significance was determined by paired Student’s *t* test (*, *P* < 0.05; **, *P* < 0.01).

Next, we additionally generated a GFP-expressing UL7 adenoviral vector. As shown in Fig. S3, transduction of human CD34^+^ HPCs with Ad UL7 leads to expression and secretion of UL7. We used each of the adenoviral vectors to transduce CD34^+^ HPCs and then fluorescence-activated cell sorting (FACS) isolated a pure population of viable CD34^+^ GFP^+^ cells and determined the effect of viral miRNA or protein expression on *FOXO3a* and *BCL2L11* mRNA levels. As shown in Fig. 4A and Fig. S4, expression of miR-US5-1, miR-UL112-3p, and the control FOXO3a shRNA downregulates *FOXO3a* (*P* = 0.0001, *P* = 0.0003, *P* = 0.0001) and *BCL2L11* (*P* = 0.0002, *P* = 0.029, *P* = 0.001) mRNA levels. Consistent with its effect only on the phosphorylation status of FOXO3a, expression of UL7 did not have any impact on *FOXO3*a mRNA levels (Fig. 4A) but significantly decreased the proapoptotic gene *BCL2L11* (*P* = 0.004) (Fig. 4B). We then wanted to evaluate the effect of UL7 and HCMV miRNAs on adenovirus-induced apoptosis (39). CD34^+^ HPCs from three independent donors were transduced with the adenoviral vectors, and phosphatidylserine translocation, as measured by annexin V staining on CD34^+^ GFP^+^ HPCs using flow cytometry, was analyzed after 72 h. As shown in Fig. 4C, CD34^+^ HPCs expressing HCMV miR-US5-1 (*P* = 0.02), miR-UL112-3p (*P* = 0.0137), or pUL7 (*P* = 0.0056) were more resistant to apoptosis than Ad GFP. These data indicate that inactivation of FOXO3a by HCMV miR-US-5-1, miR-UL112-3p, and UL7 results in decreased levels of *BCL2L11* and ultimately protection from apoptosis.

10.1128/mSphere.00986-20.3 FIG S3 UL7 expression in CD34^+^ HPCs and human fibroblasts (NHDF). (A) Three independent CD34^+^ HPC donors were transduced at an MOI of 500 with Ad GFP or Ad UL7. After 48 h, cells were FACS isolated for viable GFP^+^ CD34^+^ HPCs. RNA was isolated using TRIzol, and qRT-PCR for UL7 was performed. (B) UL7 protein was detected in the supernatant of the three donors in panel A by luminescence assay. UL7 has a tag called HiBiT, a small 11-amino-acid peptide that binds with high affinity to another larger subunit called LgBiT. The bound complex has luciferase activity and will release luminescent signal in the presence of added furimazine substrate. (C) NHDF were transduced at an MOI of 250 with Ad GFP or Ad UL7 for 48 h, proteins were subjected to immunoblotting and UL7 was detected using the Nano-Glo HiBiT blotting system, according to the manufacturer’s protocol (Promega). Download FIG S3, EPS file, 1.7 MB. Copyright © 2021 Hancock et al. 2021 Hancock et al. This content is distributed under the terms of the Creative Commons Attribution 4.0 International license.

10.1128/mSphere.00986-20.4 FIG S4 HCMV miR-US-5-1 and miR-UL112-3p expression in CD34^+^ HPCs. Three independent CD34^+^ HPC donors were transduced at an MOI of 500 with Ad GFP, Ad miR-US5-1, or Ad miR-UL112-3p. After 48 h, cells were FACS isolated for viable GFP^+^ CD34^+^ HPCs. RNA was isolated using TRIzol, and qPCR to detect miR-US5-1 and miR-UL112 was performed. Download FIG S4, EPS file, 1.1 MB. Copyright © 2021 Hancock et al. 2021 Hancock et al. This content is distributed under the terms of the Creative Commons Attribution 4.0 International license.

### UL7, miR-US5-1, and miR-UL112-3p decrease *BCL2L11* gene expression in HCMV-infected CD34^+^ HPCs.

**FIG 5:**
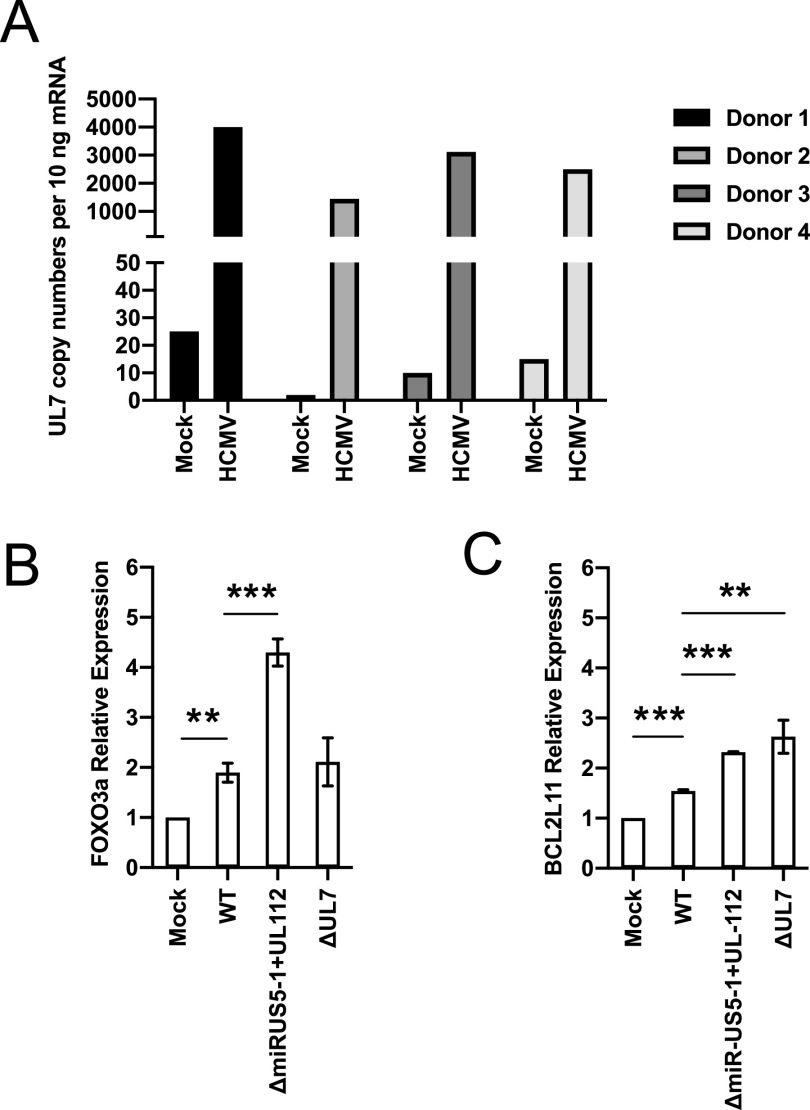
Effect of UL7, miR-US5-1, and UL112-3p deletion on FOXO3a and BCL2L11 expression in HCMV-infected CD34^+^ HPCs. (A) CD34^+^ HPCs were infected with WT TB40E for 48 h and then FACS isolated for viable CD34^+^ GFP^+^ HPCs. RNA was isolated using TRIzol, and qRT-PCR for UL7 was performed. (B and C) CD34^+^ HPCs were infected with WT TB40E, ΔiR-US5-1+UL112-3p, or ΔUL7 for 48 h and then FACS isolated for viable CD34^+^ GFP^+^ HPCs. RNA was isolated using TRIzol, and qRT-PCR for *FOXO3a* (B) and *BCL2L11* (C) was performed. Values are means ± standard errors of the means (SEM) (error bars). Statistical significance was determined by unpaired Student’s *t* test (*n* = 4; *, *P* < 0.05; **, *P* < 0.005; ***, *P* < 0.0005).

FOXO3a is mostly found in the nucleus of primitive hematopoietic cells and progenitor cells, as shown in Fig. 2C. We hypothesized that UL7, miR-US5-1, and miR-UL112-3p could act together to reduce FOXO3a levels and activity at early times postinfection to block the induction of the proapoptotic transcript *BCL2L11*. As shown in Fig. 5, UL7 is expressed at 2 days after wild-type HCMV infection in four independent donors. The expression of HCMV miR-US5-1 and miR-UL112-3p in CD34^+^ HPCs at the same time point has previously been reported (40). After sorting for a pure population of GFP^+^ CD34^+^ HPCs at 2 days postinfection (dpi), we observed that FOXO3a transcript was significantly increased in TB40EΔmiR-US5-1+UL112-3p compared to wild-type infection (*P* = 0.0003, Fig. 5B), while *BCL2L11* mRNA levels were higher in both TB40EΔmiR-US5-1+UL112-3p (*P* < 0.0001) and TB40EΔUL7 (*P* = 0.008) (Fig. 5C). These results are consistent with the hypothesis that the virus is inactivating FOXO3a using both viral miRNAs and a viral protein to prevent expression of the proapoptotic gene *BCL2L11* and to promote survival of HCMV-infected CD34^+^ HPCs.

## DISCUSSION

In the present study, we report a novel observation whereby HCMV pUL7 together with miR-US5-1 and miR-UL112-3p mediates the downregulation and inactivation of the transcription factor FOXO3a prior to establishment of latency in CD34^+^ HPCs. These data implicate multiple HCMV gene products in regulation of FOXO3a, suggesting that modulation of FOXO3a function is critical during HCMV infection of CD34^+^ HPCs. Indeed, we found that inactivation of FOXO3a leads to downregulation of the proapoptotic gene *BCL2L11* and protection from apoptosis.

Jonsson et al. previously reported that Flt-3L induces AKT/PKB phosphorylation, which inactivates FOXO3a, leading to the survival of a murine progenitor cell line stably expressing Flt-3R (FDC-P1/flt3) (33). We also observed that Flt-3L promotes FOXO3 phosphorylation via AKT signaling (Fig. 1A and B) and inactivation of the transcription factor (Fig. 1C) in telomerized human fibroblasts overexpressing the Flt-3R. Interestingly, we also observed that pUL7 promotes a time-dependent phosphorylation of FOXO3a via the Flt-3R (Fig. 1A and B) and nucleus-to-cytoplasm translocation (Fig. 1E); however, the preferential intracellular signaling pathway stimulated by pUL7 is ERK/MAPK (Fig. 1C). Specifically, pUL7 stimulation induces phosphorylation of FOXO3a on Ser294 (Fig. 1D and Fig. 2A), which along with Ser344 and Ser425 is a residue of FOXO3a that is phosphorylated by ERK. ERK-mediated phosphorylation induces FOXO3a degradation via the murine double minute-2 (MDM2) signaling pathway, which consequently stimulates cell survival and antiapoptotic gene expression (34). Indeed, we demonstrated that phosphorylation of FOXO3a by pUL7 leads to nuclear exclusion in RS4;11 cells (Fig. 2C) and in CD34^+^ HPCs (Fig. 2E) as well as downregulation of the proapoptotic gene *BCL2L11* (Fig. 2F). Besides the regulation of FOXO3a through phosphorylation, we found that HCMV encodes two miRNAs that are capable of downregulating endogenous FOXO3a mRNA and protein. Indeed, viral miRNAs regulate numerous cellular and viral processes critical for efficient viral replication including immune evasion, proinflammatory cytokine production and signaling, cell survival, and virion assembly compartment formation (reviewed in reference 8). We previously published that HCMV miR-US5-1 and miR-UL112-3p target the IκB kinase (IKK) complex components IKKα and IKKβ to limit production of proinflammatory cytokines in response to interleukin 1β (IL-1β) and tumor necrosis factor alpha (TNF-α) (41). In this study, we show that the same two HCMV miRNAs target the 3′ UTR of FOXO3a (Fig. 3A), downregulating endogenous RNA and protein (Fig. 3B to F). In hematopoietic cells, cellular miRNAs from the miR-212/132 cluster regulate FOXO3a mRNA and protein levels. Both deletion and overexpression of this miRNA cluster result in altered hematopoiesis during aging, suggesting that precise FOXO3a regulation via miRNA expression in the bone marrow is a critical component of hematopoiesis (42).

An understanding of the role of FOXO transcriptional factors in the biology of herpesviruses remains incomplete. We know from the literature that Epstein-Barr virus (EBV) latent membrane protein 1 inactivates FOXO3a via the PI3K/AKT pathway, leading to expression of miR-21 (43) and upregulation of MCL-1, both of which reduce apoptosis (44). Similarly, the LANA2 protein from Kaposi’s sarcoma-associated herpesvirus (KSHV) functionally interacts with FOXO3a and inhibits the transactivation of *BCL2L11* promoter (45). Chemical inhibition or knockdown of the related FOXO1 protein has been shown to increase the intracellular reactive oxygen species (ROS) level that is sufficient to disrupt KSHV latency and promote viral lytic reactivation (46). A recent publication by Hale et al. (47) showed an important role for FOXO proteins in HCMV reactivation from latency. FOXO1 and FOXO3a transcription factor activity is essential for inducing HCMV reactivation by activating the alternative major immediate early promoters (MIEPs) iP1 and iP2. This study builds on the observation that IE protein expression following reactivation stimuli is dependent not on expression from the MIEP but predominantly on expression from the novel internal promoters (47, 48). Thus, it appears that herpesviruses utilize multiple different mechanisms to regulate FOXO protein function to persist in infected cells and in some instances to promote viral reactivation. Our data are similar to what has been reported for KHSV LANA2 protein (45). Indeed, we found that UL7, miR-US5-1, and miR-UL112-3p inactivation of FOXO3a is important to promote survival of CD34^+^ HPCs when they are independently expressed (Fig. 4C) as well as in the context of the viral infection, as demonstrated by the increased expression of *BCL2L11* during infection with the HCMVΔmiR-US5-1+UL112-3p and ΔUL7 mutant viruses (Fig. 5D).

HCMV is known to promote an antiapoptotic response through the activation of signaling pathways in order to ensure long-term survival in nonpermissive cells (reviewed in reference 17). Indeed, the protective phenotype upon entry in CD34^+^ HPCs is achieved by virus-induced ERK-MAPK signaling that leads to the degradation of proapoptotic proteins PUMA and BIM, alongside an upregulation of MCL-1 levels (20, 21). Our findings show that the ERK-MAPK pathway is important for the UL7-mediated phosphorylation and inactivation of FOXO3a, resulting in downregulation of BIM transcript and protein and ultimately in protection from apoptosis (Fig. 4C). The role of HCMV miRNAs in protecting nonpermissive cells from apoptosis is unknown. Here, we report that HCMV miR-US5-1 and miR-UL112-3p protect CD34^+^ HPCs from apoptosis by targeting FOXO3a and ultimately decreasing *BCL2L11* gene expression. Among herpesviruses, EBV and KHSV are known to use viral miRNAs as a tool to avoid apoptosis both at early stages of infection and upon cell transformation. EBV-miR-BART5-5p regulates proapoptotic PUMA, while ebv-miR-BART-cluster 1 and ebv-miR-BART12 target BIM (49, 50). BCL2-associated death promoter (BAD) protein is downregulated by ebv-miR-BART20-5p (51). Finally, proapoptotic protein caspase-3 was identified as being a target of ebv-miR-BART16 and ebv-miR-BART1-3p (52). KHSV encodes three viral miRNAs, kshv-miR-K12-1, kshv-miR-K12-3, and kshv-miR-K12-4-3p, that cooperatively suppress caspase-3 expression and reduce apoptosis in infected cells (53).

This study illustrates for the first time a synergy between a viral gene and two miRNAs in regulating a cellular target, FOXO3a, to promote CD34^+^ HPC survival. Greater understanding of how UL7, miR-US5-1, and miR-UL112-3p work together in the context of viral infection is important to better define their role in establishment of HCMV latency and viral reactivation.

## MATERIALS AND METHODS

### Cells, reagents, and viruses.

CD34^+^ HPCs were isolated from fetal liver tissue obtained from Advanced Bioscience Resources. The tissue was manually disrupted and then digested with DNase, collagenase, and hyaluronidase, and CD34^+^ HPCs were isolated using magnetic beads (Miltenyi Biotech) as previously described (54). Cells for HCMV infection were cultured in Iscove’s modified Dulbecco’s medium (IMDM) (HyClone) supplemented with 10% BIT 9500 serum substitute (Stemcell Technologies), 50 μM 2-mercaptoethanol, 2 nM l-glutamine, 20 ng/ml human low-density lipoprotein (MilliporeSigma) as previously described (55). Human bone marrow CD34^+^ HPCs were obtained from Stemcell Technologies and recovered in SFEM II (Stemcell Technologies) supplemented with 10% BIT 9500 serum substitute. RS4;11 (bone marrow lymphoblast) cells were obtained from the American Type Culture Collection. RS4;11 cells were grown in RPMI 1640 (HyClone) medium supplemented with 10% fetal bovine serum (FBS) (HyClone), 4.5 g/liter glucose, l-glutamine and sodium pyruvate, and antibiotics (penicillin [10 U/ml]-streptomycin [10 μg/ml]). Telomerized human fibroblasts (THF) were a gift from Victor DeFilippis (Oregon Health & Science University [OHSU]). THF cells were cultured with Dulbecco’s modified Eagle’s medium (DMEM) (Cellgro) supplemented with 10% FBS, penicillin, streptomycin, and l-glutamine. Normal human dermal fibroblasts (NHDF) were cultured under conditions identical to the THF cells. Human embryonic kidney 293 cell line (HEK293) (Microbix Biosystems Inc.) cells were cultured in minimum essential medium (MEM) (Cellgro) supplemented with 10% FBS, penicillin, streptomycin, and l-glutamine. Stably transduced THF expressing human Flt-3R were generated by transduction with the lentivirus pLenti-FLT3-mGFP-P2A-Puro (OriGene Technologies RC211459L4V) followed by GFP purification after 1 week of Puro (800 μg/ml) selection. Recombinant UL7 protein from TB40E (TB40E: EF999921; UL7 protein: ABV71537.1) was generated at RayBiotech as previously described (31). Recombinant mouse IgG Fc and recombinant human Flt-3L were purchased at R&D Systems. Quizartinib (AC220), PD98059, LY294002, and LY303511 inhibitors were purchased at Selleckchem and resuspended in dimethyl sulfoxide (DMSO). HCMV TB40, HCMV TB40ΔUL7, and HCMV TB40ΔmiR-US5-1ΔmiR-UL112-3p stocks and titers were generated as previously described (31, 41).

### Immunofluorescence.

CD34^+^ HPCs from 3 independent donors were thawed and plated in StemSpan SFEM II medium (Stemcell Technologies) containing 10% BIT serum substitute in a 5% CO_2_ humidified incubator and recovered overnight (15 to 18 h) at 37°C. A total of 50,000 cells were seeded per well in an 8-well chamber slide (Thermo Scientific) in fresh SFEM II medium without additional supplements. The HPCs were then either unstimulated (mock) or stimulated for 6 h with 50 ng/ml of recombinant human Flt-3L or 50 ng/ml of recombinant UL7 protein. HPCs were fixed in 4% methanol-free formaldehyde for 15 min and then washed 3 times in phosphate-buffered saline (PBS). After permeabilizing in PBS containing 0.1% Triton X-100 for 10 min and washing in PBS, the HPCs were blocked for 1 h in freshly prepared 5% normal goat serum with 0.3% Triton X-100 in PBS. After washing in PBS, the HPCs were incubated overnight at 4°C in the primary antibody rabbit–anti-FoxO3a (Cell Signaling) or mouse–anti-CD34 (BioLegend), all diluted 1:100 in antibody dilution buffer (PBS containing 1% bovine serum albumin [BSA] and 0.3% Triton X-100). The HPCs were washed twice in PBS and then incubated 1 h in the secondary antibody goat–anti-rabbit AF594 (Invitrogen) and goat–anti-mouse AF488 (Invitrogen) diluted at 1:1,000 in antibody dilution buffer. The HPCs were mounted under a coverslip with DAPI-Fluoromount-G clear mounting medium (SouthernBiotech) and imaged using an EVOS-FL fluorescence microscope with a 100× objective. The image analysis was performed using ImageJ.

### Adenoviruses.

HCMV UL7 containing an HiBiT sequence (5′-GTGAGCGGCTGGCGGCTGTTCAAGAAGATTAGC-3′) after the signal peptide (nucleotide 142) or the regions approximately 100 bp up- and downstream of miR-US5-1 and miR-UL112 was cloned into the shuttle vector pAdTrack-CMV and transformed into TOP10 cells. After 24 h of incubation at 37°C under kanamycin selection, colonies were selected and screened for the insert by restriction enzyme digest using KpnI and XhoI. The pAdTrack plasmids were then linearized by digesting with restriction endonuclease PmeI and subsequently recombined into Escherichia coli BJ5183 cells containing the adenoviral backbone plasmid pAdEasy-1 (AdEasier-1 cells). pAdTrack-CMV (Addgene plasmid catalog no. 16405) and AdEasier-1 cells (Addgene catalog no. 16399) were a gift from Bert Vogelstein (56). Recombinants were selected for kanamycin resistance, and the recombination was confirmed by restriction endonuclease analyses. Finally, the recombinant plasmids were linearized with PacI before transfection with Lipofectamine 2000 (ThermoFisher) into the adenovirus packaging cell line HEK293. The control vector Ad GFP, Ad miR-UL112-3p, Ad miR-US5-1, and Ad UL7 were produced and purified and their titers were determined in HEK293 cells, as previously described (27). The Ad-GFP-U6-h-FOXO3a-shRNA (shADV-209273) was purchased at Vector Biosystems, Inc.

### qRT-PCR.

Total RNA was isolated from infected, transfected, or treated cells using the TRIzol RNA isolation method following the manufacturer’s directions. cDNA was prepared using 10 to 1,000 ng of total RNA and random hexamer primers. Samples were incubated at 16°C for 30 min, 42°C for 30 min, and 85°C for 5 min. Real-time PCR (TaqMan) was used to analyze cDNA levels in transfected or infected samples. An ABI StepOnePlus real-time PCR machine was used with the following program for 40 cycles: 95°C for 15 s and 60°C for 1 min. FOXO3a, BCL2L11, and 18S primer/probe sets were obtained from ThermoFisher Scientific. Relative expression was determined using the threshold cycle (ΔΔ*C_T_*) method using 18S as the standard control. For UL7 expression we used the following set of primers and probe: UL7_F primer 5′-ACTACGTGTCGTCGCTGGATT-3′, UL7_R primer 5′-ACAACTTCCACCACCCCATAAT, and UL7 probe 6-carboxyfluorescein (FAM)-CATGGCCTTGGTAGGTG-MGBNFQ. qRT-PCR assays for miR-US5-1 and miR-UL112 were purchased from ThermoFisher Scientific.

### Luciferase reporter assay.

The putative 3′ UTR of FOXO3a was cloned into the dual luciferase reporter pSiCheck2 (Clontech) using the following primers: FOXO3a F, GGCAAGGCAGCACAAAACAG; FOXO3a R, GCTTTATTTACATGCGTCACC. Site-directed mutagenesis was performed using the QuikChange PCR method. To mutate the potential miR-US5-1 site within the FOXO3a 3′ UTR, the following primers were used: FOXO3a SDM 5-1 F, CATTTTAAAAATTCAGAACTCCTGTTAATGGGAGG; FOXO3a SDM 5-1 R, CCTCCCATTAACAGGAGTTCTGAATTTTTAAAATG. To mutate the potential miR-UL112 site within the FOXO3a 3′ UTR, the following primers were used: FOXO3a SDM 112 F, CACATTTTAAAAATTCAGAACTCCTGTTAATGGGAGGATC; FOXO3a SDM 112 R, GATCCTCCCATTAACAGGAGTTCTGAATTTTTAAAATGTG. 293T cells seeded into 96-well plates were cotransfected in triplicate with 100 ng of plasmid and 100 fmol of miRNA mimic (custom designed; IDT) using Lipofectamine 2000 (Invitrogen). Cells were incubated overnight and then harvested for luciferase assay using the Dual-Glo reporter assay kit (Promega) according to the manufacturer’s protocol. Luminescence was detected using a Veritas microplate luminometer (Turner Biosystems).

### Immunoblotting.

Cytoplasmic and nuclear extracts were obtained using the NE-PER Nuclear and Cytoplasmic Extraction kit (Pierce Biotechnology). Total protein extracts were prepared in cell lysis buffer (20 mM Tris-HCl, pH 7.5, 150 mM sodium chloride [NaCl], 1% [vol/vol] polyethylene glycol octyl phenol ether [Triton X-100], 2.5 mM sodium pyrophosphate, 1 mM EDTA, 1% [wt/vol] sodium orthovanadate, 0.5 μg/ml leupeptin, 1 mM phenylmethanesulfonyl fluoride [PMSF]), run on an 8 to 12% SDS-PAGE gel, transferred to Immobilon-P transfer membranes (Millipore Corp., Bedford, MA), and visualized with antibodies specific for P-Flt3R (Tyr591, 54H1; Cell Signaling), Flt-3R (8F2; Cell Signaling), FOXO3a (Cell Signaling), P-FOXO3a (Ser253; Cell Signaling), P-FOXO3a (Ser294; Cell Signaling), P-Akt (Thr308; Cell Signaling), Akt (C73H10; Cell Signaling), ERK1 (C16; Santa Cruz), P-ERK1/2 (Thr202/Tyr204; Cell Signaling), PARP (Cell Signaling), P-6S ribosomal protein (Ser235/236, D57.2.2E; Cell Signaling), S6 ribosomal protein (5G10; Cell Signaling), BIM (C34C5; Cell Signaling), and glyceraldehyde-3-phosphate dehydrogenase (GAPDH) (Abcam). Relative intensity of bands detected by Western blotting was quantitated using ImageJ software.

### Adenovirus transduction of CD34^+^ HPCs.

CD34^+^ HPCs were freshly isolated or thawed and recovered for 3 h in IMDM containing 1% FBS, 1% penicillin-streptomycin-glutamine, and stem cell cytokines (stem cell factor [SCF], FLT3L, IL-3, and IL-6). Following recovery, cells were washed in PBS and resuspended at low volume in IMDM containing 10% BIT serum supplement, l-glutamine, low-density lipoproteins, 2-mercaptoethanol, and stem cell cytokines as previously described (55) in a low-binding 24-well plate (Corning low-attachment HydroCell). HPCs were infected with adenoviruses at a multiplicity of infection (MOI) of 500 for 4 h with continual rocking and then spin infected at 300 × *g* for 30 min, resuspended, and cultured overnight. Culture conditions were supplemented with additional media, and infection continued for a total of 48 to 72 h. Samples were then FACS isolated for pure populations of transduced HPCs (viable, CD34^+^ GFP^+^) as previously described (31) or analyzed by flow cytometry as described below.

### Flow cytometry analysis for apoptosis.

CD34^+^ HPCs were transduced with adenoviruses for 72 h, washed in PBS, and stained with fixable viability dye eFluor 780 (Invitrogen/eBioscience). Cells were washed twice in FACS buffer, blocked, and stained with surface antibodies for stem cell markers including CD34 as previously described (54). HPCs were stained with annexin V (eBioscience) according to the manufacturer’s instructions and then washed and fixed with 2% formalin as previously described (54). Data were acquired on an LSRII flow cytometer (Becton Dickinson) running FACSDiva software, and data were analyzed with FlowJo v10.7 (TreeStar).

### Statistical analysis.

Data are expressed as the mean ± standard error of the mean (SEM). Statistical analysis was performed using GraphPad Prism (v8) for comparison between groups using Student’s *t* test or one-way analysis of variance (ANOVA) with Tukey posttest as indicated. A value of *P* < 0.05 or lower was considered significant, and exact *P* values are indicated.
